# Potential Prognostic Value and Mechanism of Stromal-Immune Signature in Tumor Microenvironment for Stomach Adenocarcinoma

**DOI:** 10.1155/2020/4673153

**Published:** 2020-06-26

**Authors:** Xinying Zhu, Xiaoli Xie, Qingchao Zhao, Lixian Zhang, Changjuan Li, Dongqiang Zhao

**Affiliations:** ^1^Department of Gastroenterology, Second Hospital of Hebei Medical University, Shijiazhuang, 050000 Hebei Province, China; ^2^Department of Gastroenterology, Third Hospital of Hebei Medical University, Shijiazhuang 050051, Hebei Province, China; ^3^Department of Gastroenterology, Second Hospital of Baoding, Baoding, 071051 Hebei Province, China

## Abstract

Stomach adenocarcinoma (STAD) is one of the most common malignancies. But the molecular mechanism is unknown. In this study, we downloaded the transcriptional profiles and clinical data of 344 STAD and 30 normal samples from The Cancer Genome Atlas (TCGA) database. Stromal and immune scores of STAD were calculated by the Estimation of Stromal and Immune cells in Malignant Tumor tissues using Expression data (ESTIMATE) algorithm, and association of stromal/immune scores with tumor differentiation/T/N/M stage and survival was investigated. The differentially expressed genes (DEGs) between high and low score groups (based on media) were identified, and prognostic genes over-/underexpressed in both STAD and stromal/immune signature were retrieved. Furthermore, proportions of 22 infiltrating immune cells for the cohort from TCGA were estimated by the Cell-type Identification By Estimating Relative Subsets Of RNA Transcripts (CIBERSORT) algorithm, and association of 22 immune cells with tumor differentiation/T/N/M stage and survival was investigated. Next, coexpression analysis of 22 immune cells and intersection genes over-/underexpressed in both STAD and stromal signature was conducted. We found high stromal and immune scores and macrophage infiltration predicting poor tumor differentiation and severe local invasion, obtained a list of prognostic genes based on stromal-immune signature, and explored the interaction of collagen, chemokines such as CXCL9, CXCL10, and CXCL11, and macrophage through coexpression analysis and may provide novel prognostic biomarkers and immunotherapeutic targets for STAD.

## 1. Introduction

Stomach cancer is reported to be responsible for more than 1 000 000 new cases and approximately 783 000 deaths in 2018, making it the fifth most common cancer and the third leading cause of cancer death worldwide [1]. This malignancy includes several pathological types, and stomach adenocarcinoma (STAD) accounts for the majority. Although surgical treatment and chemotherapy have improved the clinical outcomes, unfortunately, the overall survival of STAD remains poor [1–3]. The inadequate understanding of the tumor mechanism was one of the crucial reasons; therefore, it is imperative to explore the mechanism of STAD.

Tumor microenvironment (TME) is the local biological environment of tumor cells, mainly consisting of stromal cells, extracellular matrix, and cytokines. In TME, stromal cells predominantly include cancer-associated fibroblasts, endothelial cells, and immune cells such as T lymphocytes, B lymphocytes, macrophages, dendritic cells, and neutrophils, and the extracellular matrix is usually composed of collagen fiber, elastic fiber, fibronectin, laminin, and some glycans [4]. Accumulating evidence has elucidated that stromal and immune cells in the TME also play a very essential role in tumorigenesis, progression, and clinical outcomes besides cancer cells themselves in gastric cancer [5–7]. However, the distinct mechanism remains unclear.

The variety of stromal and immune cells and the complexity of their interactions in TME bring us a great challenge for experimental research [8]. However, now, the decreasing costs of next-generation sequencing technology have been providing us numerous amounts of RNA sequencing data, and we can retrieve them from some public databases such as The Cancer Genome Atlas (TCGA) [9]. Furthermore, several algorithms have been presented to predict the composition of stromal and immune cells in TME from the transcriptome data of bulk tumors, facilitating the exploration of stromal and immune signatures in TME. In 2013, Yoshihara et al. [10] put forward a program, known as Estimation of Stromal and Immune cells in Malignant Tumor tissues using Expression data (ESTIMATE), which can infer the levels of stromal and immune cells in TME based on the gene expression data. Another computational algorithm, namely, Cell-type Identification By Estimating Relative Subsets Of RNA Transcripts (CIBERSORT, https://cibersort.stanford.edu/), was presented to estimate relative fractions of 22 immune cell subsets from gene expression profiles of tumor tissues, and its accuracy has been confirmed by flow cytometry in breast cancer and liver cancer [11]. Several prognostic models based on stromal and immune signatures by ESTIMATE or CIBERSORT algorithm have been constructed [5, 12–17], some of which were even better than TNM stage in predicting cancer prognosis [17], suggesting the efficacy of the algorithms [18], as well as the importance of TME in influencing tumor biological behavior [19, 20]. However, previous models mainly focused on the prognostic value of stromal-immune related genes in TME, without considering their coexpression relationship in gastric cancer and the differential expression between gastric cancer and normal tissues; that is to say, the mechanism of stroma and immune-related genes in TME has not been explored.

**Figure 1:**
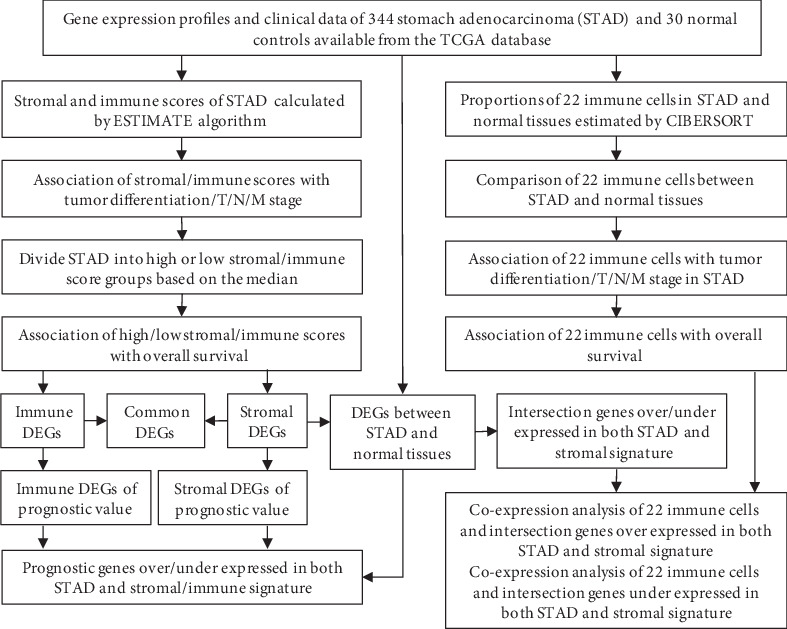
The flow diagram of the analytical process. TCGA: The Cancer Genome Atlas; ESTIMATE: Estimation of Stromal and Immune cells in Malignant Tumor tissues using Expression data; CIBERSORT: Cell-type Identification By Estimating Relative Subsets Of RNA Transcripts; DEGs: differentially expressed genes.

In the present study, we comprehensively applied the algorithms mentioned above to analyze the transcriptional and clinical data of STAD available in the TCGA database. We found that high stromal and immune scores as well as macrophage were associated with poor tumor differentiation and severe local invasion and also obtained a set of stromal and immune-related prognostic genes which are specifically expressed in STAD. Furthermore, we preliminarily explored the interaction of stromal and immune molecules in TME by coexpression analysis and may provide novel prognostic biomarkers and immunotherapeutic targets for STAD (Figure 1).

## 2. Materials and Methods

### 2.1. Data Collection

Level 3 transcriptional profiles of STAD patients and normal controls including Fragments Per Kilobase of exon per Million mapped fragments (FPKM) and counts of high-throughput sequencing mRNA, as well as the clinical data such as age, gender, overall survival, tumor differentiation grade, and TNM stage for corresponding patients, were downloaded from the TCGA database (https://tcga-data.nci.nih.gov/tcga/) on January 15, 2020.

### 2.2. Identification of Differentially Expressed Genes (DEGs) between STAD and Normal Tissue

The R package “edgeR” with a threshold of absolute value of log2 (fold change, FC) > 1 and false discovery rate (FDR) < 0.05 was used to identify the DEGs between STAD and normal tissues.

### 2.3. ESTIMATE Algorithm Analysis

Stromal and immune scores for STAD patients from TCGA were calculated by the ESTIMATE algorithm; then, the relationship of stromal and immune scores with clinicopathological characteristics was analyzed by the Wilcoxon test (two groups) or Kruskal test (more than two groups), respectively. *P* < 0.05 was regarded as statistically significant. Based on the median score, all of the STAD samples were assigned into high or low stromal/immune score groups, and the relationship of stromal/immune scores with overall survival was analyzed by the “survival” R package. In addition, the stromal DEGs between high and low stromal score groups were identified by the “limma” R package with absolute value of log2 FC > 1 and FDR < 0.05. The immune DEGs were obtained through the same methods. DEGs overexpressed in the high stromal/immune score group compared with the low score group were regarded as “overexpressed” in stromal/immune signature, while those underexpressed in the high score group were regarded as “underexpressed.” Furthermore, a Venn diagram was used to identify the common DEGs shared by stromal and immune signatures. To explore the function of the stromal and immune DEGs, as well as the common DEGs, Gene Ontology (GO) [21] and Kyoto Encyclopedia of Genes and Genomes (KEGG) [22] enrichment was performed with adjusted *P* < 0.05 as statistically significant, and a protein-protein interaction (PPI) network was constructed in STRING (https://string-db.org) with confidence > 0.95.

### 2.4. Identification of Prognostic Genes Associated with Stromal and Immune Signatures in STAD

According to the median expression level of each stromal DEGs, all STAD patients were divided into high or low expression group, and the correlation of high/low expression with survival was analyzed by the Kaplan-Meier curve and log-rank test. *P* < 0.05 was considered to be statistically significant. The stromal DEGs of prognostic value were acquired. Subsequently, these prognostic genes over- or underexpressed in stromal signature were intersected with over- or underexpressed DEGs in STAD, respectively, so we got the prognostic genes over-/underexpressed in both STAD and stromal signature. In the same way, the prognostic DEGs over-/underexpressed in both STAD and immune signature were obtained.

### 2.5. Validation of Prognostic Genes of Stromal/Immune DEGs for STAD in GEO Database

The GES84433 STAD transcriptional profiles and survival data were downloaded from the GEO database (https://portal.gdc.cancer.gov), and the ESTIMATE algorithm was used to calculate the stromal scores and immune scores of all STAD samples. According to the method of the TCGA database, we obtained the stromal/immune DEGs, from which genes with prognostic value were screened out.

### 2.6. CIBERSORT Algorithm Analysis

Proportions of 22 infiltrating immune cell subsets for 344 STAD patients and 30 normal controls from the TCGA database were estimated by the CIBERSORT algorithm with LM22 signature and 1 000 permutations, and cases with CIBERSORT *P* < 0.05 were selected for further analysis.

Comparison of 22 immune cell subsets between STAD and normal controls was conducted by the Wilcoxon test. The correlation of 22 immune cells with the clinicopathological characteristics was analyzed by the Wilcoxon test (two groups) or Kruskal test (more than two groups). Moreover, according to the median infiltrating level of each immune cell, all tumor samples were assigned into high or low infiltrating groups; Kaplan-Meier curves and log-rank test were used to evaluate the association of immune cell infiltrating levels and overall survival. *P* < 0.05 was considered statistically significant.

### 2.7. Coexpression Analysis

The shared genes overexpressed in both STAD and stromal signature were taken by intersection, and so did the genes underexpressed in both. Subsequently, the coexpression analysis of 22 infiltrating immune cells and over-/underexpressed intersection genes was performed via Pearson correlation analysis.

R version 3.6.3 was used for all analyses and plots.

## 3. Results

### 3.1. Data Preparation

Transcriptional expression profiles and clinical data of 344 STAD patients and 30 normal controls were downloaded from the TCGA database. Among the 344 STAD patients, 127 (36.9%) cases were female and 217 (63.1%) were male, with the age ranging from 35 to 90 years. The data included 8 cases (2.3%) of G1, 128 cases (37.2%) of G2, 200 cases (58.1%) of G3, and 8 cases (2.3%) of unknown differentiation grade. There were 50 (14.5%), 103 (29.9%), 135 (39.2%), and 34 (9.9%) cases classified from stage I to stage IV and 22 cases (6.4%) of unknown stage, including 19 (5.5%), 74 (21.5%), 158 (45.9%), and 85 (24.7%) cases from stages T1 to T4 and 8 (2.3%) cases of unknown T stage; 103 (29.9%), 89 (25.9%), 71 (20.6%), and 65 (18.9%) cases from stages N0 to N3 and 16 (4.7%) cases of unknown N stage; and 305 (88.7%) cases for M0, 23 (6.7%) for M1 stage, and 16 (4.7%) cases of unknown M stage.

### 3.2. Identification of Differentially Expressed Genes (DEGs) between STAD and Normal Tissues

A total of 7275 overexpressed and 3647 underexpressed DEGs were identified in STAD compared with normal tissues with the threshold of absolute value of log2 (fold change, FC) > 1 and false discovery rate (FDR) < 0.05.

### 3.3. Association of Stromal and Immune Scores with Clinicopathological Characteristics and Prognosis in STAD

**Figure 2:**
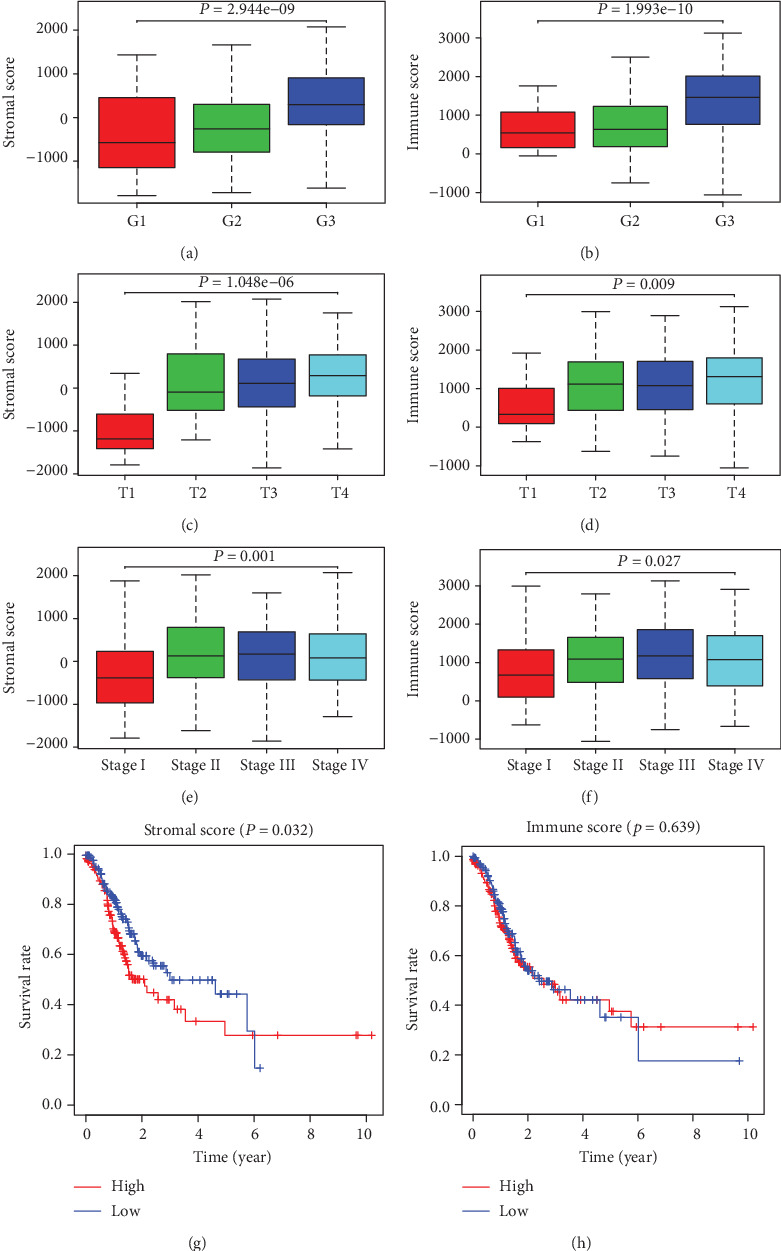
Association of stromal and immune scores with clinicopathological characteristics and prognosis in STAD. The stromal and immune scores of 344 STAD samples were calculated by the ESTIMATE algorithm. The correlation analyses revealed that increased stromal and immune scores were significantly associated with poor tumor differentiation (a, b), advanced tumor invasion depth (c, d), and stages (e, f). According to the median, all STAD patients were divided into high or low stromal/immune score groups. Survival curves demonstrated that high stromal scores predicted poor overall survival (g), while there was no significant correlation between immune scores and prognosis (h).

The stromal and immune scores of 344 STAD samples were calculated by the ESTIMATE algorithm, ranging from -1859.572772 to 2072.412073 for stromal scores and -1056.13449 to 3124.523063 for immune scores, respectively. The correlation analyses of stromal/immune scores with clinicopathological characteristics revealed that increased stromal and immune scores were significantly associated with poor tumor differentiation (Figure 2(a), *P* = 0.001; Figure 2(b), *P* = 0.001) and advanced local invasion and stages (Figure 2(c), *P* < 0.001; Figure 2(d), *P* < 0.001; Figure 2(e), *P* = 0.001; and Figure 2(f), *P* = 0.027) and that neither stromal nor immune scores were correlated with lymph nodes or distant metastasis.

To investigate the potential association of stromal/immune scores with prognosis, all STAD patients were classified into high or low stromal/immune score groups according to the median score. Survival curves demonstrated that high stromal scores predicted poor overall survival (Figure 2(g), *P* = 0.032), while there was no significant correlation between immune scores and prognosis (Figure 2(h), *P* = 0.639).

### 3.4. Identification and Function Analysis of Stromal and Immune DEGs in STAD

**Figure 3:**
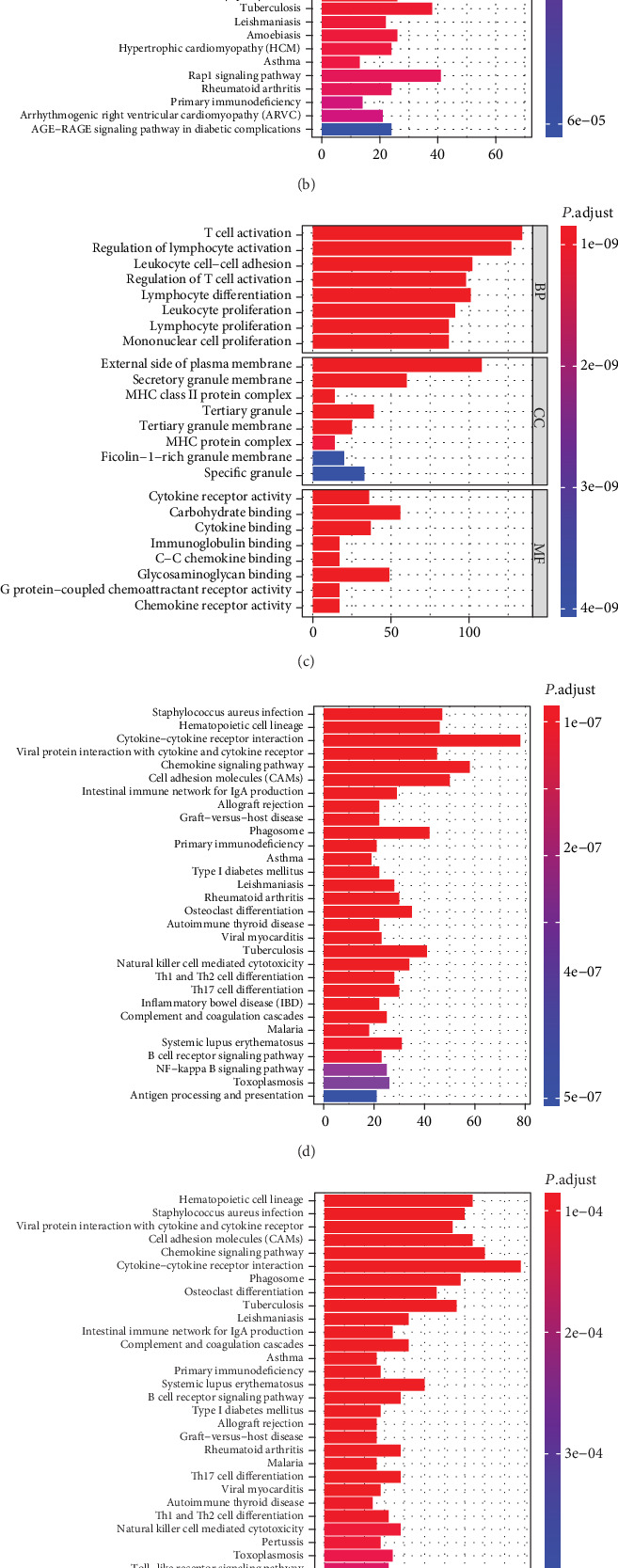
Function analysis of stromal DEGs, immune DEGs, and common DEGs in STAD. GO and KEGG enrichment of stromal DEGs (a, b). GO and KEGG enrichment of immune DEGs (c, d). KEGG enrichment (e) and the top 30 remarkable nodes retrieved from the PPI network (f) for common DEGs.

In total, 344 STAD patients were assigned into high or low stromal score groups based on the median score. Stromal DEGs were identified with absolute value of log2 FC > 1 and FDR < 0.05 as a threshold, and 1508 overexpressed and 216 underexpressed DEGs were retrieved. In the same way, a total of 861 overexpressed and 309 underexpressed immune DEGs were obtained. In order to elucidate the potential functions of the DEGs, Gene Ontology (GO) and Kyoto Encyclopedia of Genes and Genomes (KEGG) enrichment was performed. For stromal DEGs, GO annotations predominantly involved the extracellular structure and matrix organization, leucocyte activation and migration for biological processes (BP), and collagen-containing extracellular matrix for cellular component (CC) (Figure 3(a)), and KEGG enrichment mainly included cytokine-cytokine receptor interaction and PI3K-Akt signaling pathway (Figure 3(b)). For immune DEGs, GO annotations predominantly included activation, differentiation, and proliferation of lymphocytes (Figure 3(c)), while KEGG enrichment mainly involved cytokine-cytokine receptors, chemokine signaling pathways, and cell adhesion molecules (Figure 3(d)). In addition, protein-protein interaction (PPI) networks of stromal and immune DEGs were constructed in STRING, and the top 30 hub genes with high connectivity in the module were obtained (Supplementary Figures 1A and 1B).

Moreover, the Venn diagram was used to identify the common DEGs shared by stromal and immune signatures, and 644 overexpressed and 121 underexpressed genes were obtained (Supplementary Figure 1C). Predominant GO enrichment terms of the 765 common DEGs included activation, proliferation and differentiation of immune cells and their regulations, lymphocyte cell-cell adhesion, and heparin, cytokine, and chemokine binding (Supplementary Figure 1D), while the principal KEGG enrichment pathway involved cytokine-cytokine receptor interaction, chemokine signaling pathway, and cell adhesion molecules (CAMs) (Figure 3(e)). Furthermore, the PPI network was constructed (Supplementary Figure 1E), from which the top 30 remarkable nodes were acquired (Figure 3(f)), predominately containing chemokine and chemokine receptor families such as *CXCL 9*, *CXCL 10*, *CXCL 11*, *CXCL 13*, *CCL 4*, *CCL 5*, *CCL 13*, *CCL 19*, *CCL 21*, *CCR1*, *CCR 2*, *CCR 5*, *CCR 7*, *CXCR 3*, and *CXCR 4*, followed by T cell surface glycoprotein. Integrin subunits such as *ITGAM* and *ITGB2*, as well as complement 3 (*C3*), were also involved in the top 30 genes.

### 3.5. Identification of Prognostic Genes Associated with Stromal and Immune Signatures in STAD

The Kaplan-Meier curve and log-rank analysis (*P* < 0.05) revealed 263 prognostic genes from stromal DEGs, including 251 overexpressed and 12 underexpressed genes. Subsequently, these prognostic genes were intersected with 7275 overexpressed and 3647 underexpressed DEGs in STAD, respectively; then, we got 29 prognostic genes overexpressed in both STAD and stromal signature (Supplementary Figure 2A1-A29) and two prognostic genes underexpressed in both (Supplementary Figure 2B1-B2). In the same way, 63 immune DEGs of prognostic value were identified, containing 41 overexpressed and 22 underexpressed genes. Next, five prognostic DEGs overexpressed in both STAD and immune signature (Supplementary Figure 2B1-B2, D1-D2) and four underexpressed in both (Supplementary Figure 2A27-A29, C1-C2) were obtained.

### 3.6. Validation of Prognostic Genes of Stromal/Immune DEGs for STAD in GEO Database

Based on GES84433 data consisting of 357 STAD patients from the GEO database, the relationship between stromal/immune scores obtained by the ESTIMATE algorithm and overall survival showed that high stromal scores were significantly correlated with poor prognosis in STAD patients (Supplementary Figure 3A, *P* = 0.049), while no significant correlation was found between immune scores and prognosis (Supplementary Figure 3B, *P* = 0.089). There were 159 genes with prognostic value in stromal-related DEGs in GEO data, and 41 shared prognostic genes with 263 stromal-related DEGs in TCGA data (Supplementary Figure 3C). There were 65 prognostic genes in immune-related DEGs in GEO data including 26 shared prognostic genes with 63 immune-related DEGs in TCGA data (Supplementary Figure 3D).

### 3.7. Landscape of 22 Infiltrating Immune Cells by CIBERSORT Algorithm

**Figure 4:**
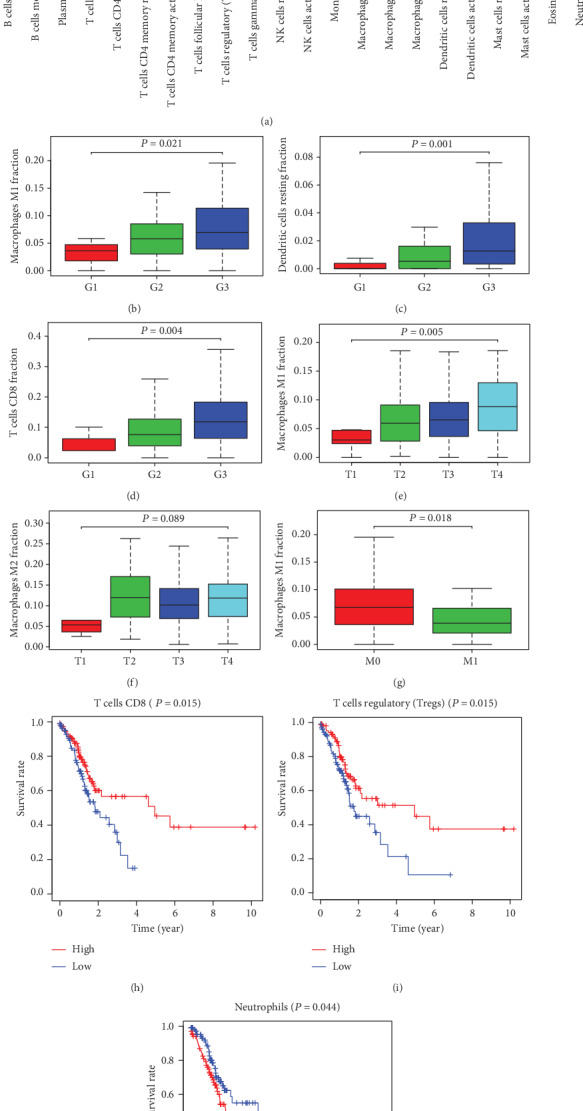
Correlation of 22 immune cells with clinicopathological characteristics and prognosis in STAD. Comparison of 22 immune cells between STAD and normal controls (a). Infiltrating immune cells related to the tumor differentiation grade (b–d), the local tumor invasion (e, f), the distant metastasis (g), and the prognosis of STAD (h–j).

Proportions of 22 infiltrating immune cells for 344 STAD and 30 normal samples were estimated by the CIBERSORT algorithm, and 221 STAD and 15 normal cases with CIBERSORT *P* < 0.05 were selected for further analysis. The landscape of 22 immune cells in STAD and controls was displayed by a bar plot and heat map (Supplementary Figure 4). Comparison of 22 immune cell subsets between tumor and control sample was exhibited by a violin plot (Figure 4(a)), demonstrating that the proportions of macrophages M0, M1, and M2 significantly increased (all *P* < 0.001), while plasma cells decreased (*P* < 0.001) in STAD.

### 3.8. Correlation of 22 Immune Cells with Clinicopathological Characteristics and Prognosis in STAD

The correlation analyses of 22 immune cells with the tumor differentiation grade, local invasion degree, lymph node metastasis, and distant metastasis showed that high proportions of macrophage M1, dendritic cells, and CD8 T cells predicted poor tumor differentiation (Figure 4(b), *P* = 0.021; Figure 4(c), *P* = 0.001; and Figure 4(d), *P* = 0.004), high macrophage M1 infiltration indicated advanced tumor invasion (Figure 4(e), *P* = 0.005), and macrophage M2 tended to be positively correlated with severe tumor invasion, but *P* = 0.089 (Figure 4(f)). Moreover, high macrophage M1 infiltration was negatively related to the risk of distant metastasis (Figure 4(g), *P* = 0.018), while no significant association was observed between macrophage M2 and distant metastasis (*P* = 0.759). There was no significant correlation between the proportions of 22 immune cells and lymph node metastasis.

The Kaplan-Meier curve and log-rank analysis were used to analyze the relationship between 22 immune cells and overall survival rates, revealing that elevated proportions of CD8 T cells and regulatory T cells (Tregs) predicted good prognosis (Figure 4(h), *P* = 0.015; Figure 4(i), *P* = 0.015), while increased neutrophils were related to poor prognosis (Figure 4(j), *P* = 0.044). Moreover, high proportions of plasma cells tended to have a good prognosis, but *P* = 0.083. According to the CIBERSORT algorithm, there was no significant association between macrophages M0, M1, and M2 and the overall survival rate (*P* = 0.815, *P* = 0.989, and *P* = 0.317).

### 3.9. The Coexpression Analysis

**Figure 5:**
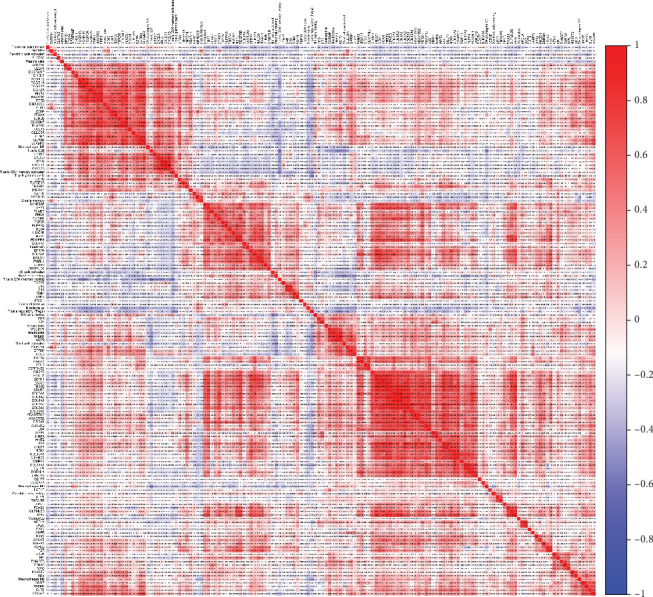
The coexpression analysis of 22 infiltrating immune cells and overexpressed intersection genes in both STAD and stromal signature.

**Figure 6:**
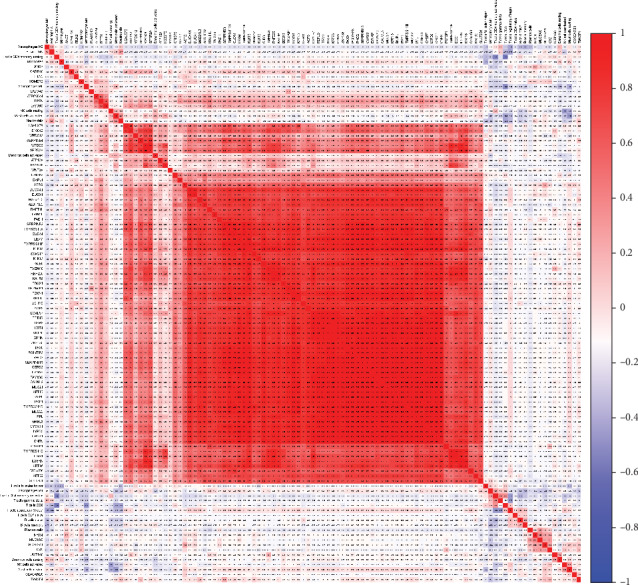
The coexpression analysis of 22 infiltrating immune cells and underexpressed intersection genes in both STAD and stromal signature.

A total of 132 intersection genes overexpressed in both STAD and stromal signature and 87 intersection genes underexpressed in both were identified. Subsequently, the coexpression analysis of 22 infiltrating immune cells and over-/underexpressed intersection genes was exhibited by heat maps (Figures 5 and 6).

## 4. Discussion

In recent years, TME has been reported to play an essential role in tumorigenesis, progression, metastasis, therapeutic response, and prognosis and has become a new research hotspot with the development of immunotherapy and precision treatment in malignancies [19, 23]. Meanwhile, STAD has been regarded as a public health problem worldwide due to the high morbidity and mortality [1, 2]. Thus, our study focused on the TME in STAD.

Our results obtained by the ESTIMATE algorithm showed that a high stromal score indicated poor prognosis, which was consistent with a previous study of Wang et al. [14]. Moreover, in the study of Jiang et al. [17], formalin-fixed paraffin-embedded specimens of 879 patients with gastric cancer after operation were included to construct the immune score model based on five immune characteristics, supporting the conclusions that the immune score was an independent prognostic factor and that the predictive value of the immune score combined with TNM on the survival rate and postoperative recurrence of gastric cancer was better than that of TNM stage alone. Therefore, the stromal-immune signature is a good supplement to traditional pathological staging.

In the present study, all of the 29 prognostic genes that are overexpressed in both STAD and stromal signature, unfortunately, indicated poor prognosis, among which there were several collagen-encoding mRNA such as *COL1A2*, *COL5A1*, *COL5A2*, *COL8A1*, *COL10A1*, and *COL12A1*. Through coexpression analysis, these collagens were found to be positively correlated with each other and with numerous important molecules such as *LOX*, *MMP11*, *FAP*, and *WNT* as well as macrophage M2, suggesting that collagen may play a crucial role of eliciting poor outcomes by cooperating with the above molecules in TME [24]. Studies have shown that the new model including the collagen signature had a better predictive value for lymph node metastasis in early gastric cancer than the traditional TNM model [25]. It can be seen that collagen, an important component of TME, should be taken seriously in STAD, whether acting as a prognostic prediction or a therapeutic target [26].

The results of the CIBERSORT algorithm in our study showed that CD8+ T and Treg infiltration were positively correlated with the overall survival rate, while neutrophil infiltration reached an inverse outcome. CD8+ T, also known as cytotoxic T cell (CTL), carries a function of antitumor immunity and promotes tumor cell apoptosis, responsible for good clinical outcomes of various malignant tumors including gastric cancer [4, 27, 28]. Treg, defined as CD4+ CD25+ Foxp3+ T cell, is capable of immunosuppression which helps tumor cells escape from the immune system, accounting for poor prognosis in a variety of solid malignancies [29]. However, heterogeneity in differentiation and phenotype among Tregs has been demonstrated [30], leading to conflicting reports of its role in tumor prognosis. According to Kim et al. [28], Treg was associated with poor prognosis in patients with microsatellite-unstable gastric cancers, whereas in the research of Liu et al. [31], Treg was an independent predictor of short overall survival in gastric cancer. A retrospective study involving 598 gastric cancer patients suggested that high infiltration of FOXP3+ Treg was of better prognosis in patients on stages I-II, whereas the converse outcome was demonstrated in that of stages III-IV [32]. Further prospective studies based on different subtypes may be required to elucidate the association of Treg with gastric cancer.

The correlation analyses of stromal and immune scores with clinicopathological characteristics of STAD revealed that both high stromal and immune scores were related to poor tumor differentiation and severe local invasion and that neither was significantly related to lymph nodes and distant metastasis, indicative of the greater influence of TME on primary tumor cells than metastasis.

TME is a complex system involving multiple cells and cytokines. Among the immune cells, macrophage, namely, tumor-associated macrophage (TAM), is particularly abundant and plays a crucial role in tumor progression. TAM exposed to cytokines such as TNF-*α*, INF-*γ*, or lipopolysaccharide (LPS) acquires M1 polarization. Conversely, some cytokines including IL4, IL10, and IL13 can promote TAM polarization toward the M2 state. Traditionally, M1 is considered to be proinflammatory and antitumor, while M2 is confirmed to be anti-inflammatory and protumor [33]. However, these perspectives seem to be absolutism. Compared with uncomplicated gastritis, enhanced M1 macrophage polarization was observed in H. pylori-associated atrophic gastritis which was regarded as a premalignant lesion of gastric cancer [34]. Another study has confirmed that at the early stages of gastric cancer, NF-*κ*B polarized macrophages toward M1 by increasing the transcription of proinflammatory factors. In contrast, at the advanced stage, macrophages were polarized toward M2 due to the functional deficit of NF-*κ*B [35]. A long-term follow-up study enrolling 1138 patients with gastric adenoma confirmed that the number of TAM was an independent risk factor for the progression of carcinoma development [36]. Our study also showed that the proportions of macrophages M1 and M2 were significantly increased in STAD compared with normal controls, the increased proportion of M1 indicated poor tumor differentiation and severe local invasion, and high proportion of M2 seemed to represent severe local invasion. Therefore, both M1 and M2 phenotypes of macrophages may promote tumor growth. Moreover, it has been confirmed that M1 and M2 phenotypes can be converted to each other [37]. The 5-year survival rate of gastric cancer at an advanced stage is less than 30% even after surgical treatment [3], while it can exceed 90% at an early stage after treatment [38]. Given that knowledge, prevention, and early diagnosis of gastric cancer are of great importance. Therefore, TAM, especially M1 phenotype, as an important initiating factor in the early stage of STAD, should be paid more attention.

There is a complex network of cytokines released by both malignancy and diverse stromal cells in TME to contribute to carcinogenesis [35]. In our study, the PPI plot of common DEGs shared by stromal and immune signatures also showed that various cytokines, especially chemokine and chemokine receptor family including *CXCL9*, *CXCL10*, *CXCL11*, *CXCL13*, *CCL4*, *CCL5*, *CCL13*, *CCL19*, *CCL21*, *CCR1*, *CCR2*, *CCR5*, *CCR7*, *CXCR3*, and *CXCR4*, were the most active and quite important cytokines in TME. Some of these chemokines and receptors, such as *CXCL13*, *CCL4*, *CCL5*, *CCR2*, *CXCR3*, and *CXCR4*, have been experimentally confirmed to be associated with the pathogenesis or prognosis of gastric cancer or other malignancies [39, 40–43]. In our study, *CXCL9*, *CXCL10*, and *CXCL11* were found to be overexpressed in STAD compared with the normal control and in the stromal signature. Moreover, there was a positive coexpression relationship among them and with the proportion of macrophage M1.

Naturally, *CXCL9*, *CXCL10*, and *CXCL11* were expressed at low levels, and they could be upregulated by cytokine stimulation. *CXCL9*, *CXCL10*, and *CXCL11* in TME were primarily secreted by tumor cells, monocytes, fibroblasts, and endothelial cells in response to IFN-*γ* [43]. The common receptor of these ligands was *CXCR3*, which had three spliced variants in human (*CXCR3A*, *CXCR3B*, and *CXCR3-alt*). *CXCR3* is usually expressed on the surface of tumor cells, monocytes, dendritic cells, T cells, and NK cells [42]. *CXCL9*, *CXCL 10*, and *CXCL11* were reported to recruit immune cells to TME, stimulate and induce immune cells such as macrophages, CD8+ T cells, and NK cells to produce TNF-*α*, IFN-*γ*, and IL-2 through Th1 polarization and activation, and thus enhance antitumor immunity. In turn, the IFN-*γ*-dependent immune activation can also promote the release of the above chemokines [41]. The *CXCL9*, *CXCL10*, and *CXCL11*/*CXCR3* axis was generally considered to have antiangiogenic effects on endothelial cells and has been reported as effective tumor angiogenesis inhibitors in some in vivo tumor models, including pancreatic cancer, breast cancer, lung cancer, and melanoma [40]. However, there have also been reports of *CXCL9*, *CXCL10*, and *CXCL11* that promote tumor proliferation in colon cancer, esophageal adenocarcinoma, and head and neck cancer by promoting inflammation or other mechanisms [44–47]. The effects of *CXCL9*, *CXCL10*, and *CXCL11* on prognosis have also been reported variously: in colon cancer, esophageal cancer, non-small-cell lung cancer, and ovarian cancer, they were reported to favor good outcomes, while in pancreatic cancer and clear cell kidney cancer, they represented poor prognosis [48]. Different variants of *CXCR3* may be one of the reasons, and other mechanisms leading to contradictory results remain unclear. There have been several attempts to target this axis for immunotherapy in colon cancer, breast cancer, kidney cancer, mesothelioma, and myeloma, but it should be treated differently in different types of tumors and subtypes [43]. However, there were few studies on the mechanism of *CXCL9*, *CXCL10*, and *CXCL11* and their relationship with macrophage in STAD, which need further exploration and may provide a new target of immunotherapy for STAD [49].

Inevitably, there are several limitations to this article. The absence of experimental validation could be the major limitation. Furthermore, the data were obtained from the TCGA database, lacking information of Asian ethnicity, and the results may not be suitable for all ethnicities. To make up for the limitations, we are preparing an international multicenter clinical study of STAD in which we will explore the mechanisms of collagen, *CXCL 9*, *CXCL10*, *CXCL11*, and TAM in TME by PCR, Western blotting, immunohistochemistry, and flow cytometry to validate our results.

## 5. Conclusions

Taken together, in this study, we analyzed the data of STAD from the TCGA database by ESTIMATE and CIBERSORT algorithms and exhibited the landscape of stromal and immune signatures in TME. We found that high stromal and immune scores and macrophage infiltration were associated with poor tumor differentiation and severe local invasion and also obtained a list of prognostic genes based on the stromal-immune signature and differentially expressed in STAD compared with normal tissues. Moreover, we preliminary explored the interaction of collagen, chemokines such as *CXCL9*, *CXCL10*, and *CXCL11*, and TAM in TME through coexpression analysis of transcriptome data associated with stromal and immune signatures and may, therefore, provide novel prognostic biomarkers and immunotherapeutic targets for STAD.

## Data Availability

All the data in the present study are available in the public database (https://portal.gdc.cancer.gov/).

## Supplementary Materials

Supplementary Materials Supplementary Figure 1: function analysis of stromal DEGs, immune DEGs, and common DEGs in STAD. Supplementary Figure 2: prognostic genes associated with stromal and immune signatures in STAD from the TCGA database. Supplementary Figure 3: validation of prognostic genes of stromal/immune DEGs for STAD in the GEO database. Supplementary Figure 4: the bar plot (A) and heat map (B) of 22 infiltrating immune cells in STAD and controls estimated by the CIBERSORT algorithm.
