# Supplementary material for: Potential Prognostic Value and Mechanism of Stromal-Immune Signature in Tumor Microenvironment for Stomach Adenocarcinoma
Source: Biomed Res Int. 2020 Jun 26;2020:4673153. doi: 10.1155/2020/4673153 (PMC7335387; doi:10.1155/2020/4673153)
Supplement: Supplementary file 1 [file 4673153.f1.odt]

722

723 Supplementary Figure 1. Function analysis of stromal DEGs, immune DEGs and

724 common DEGs in STAD.

725    The top 30 remarkable nodes in PPI network of stromal and immune DEGs (A-B).    726  Venn diagram was used to identify the common DEGs shared by stromal and immune 727 signature (C). The GO enrichment (D) and the PPI network (E) of the common DEGs. 728

729

730

731 Supplementary Figure 2. Prognostic genes associated with stromal and immune

732 signature in STAD from TCGA database.

733  There were 29 prognostic genes over expressed in both STAD  and stromal signature   734   (A1-A29),  and  two  prognostic  genes  under  expressed  in  both  (2B1-B2).  Five    735  prognostic genes were over expressed in both STAD and immune signature (2B1-B2,  736 D1-D2) and four were under expressed in both (2A27-A29, C1-C2).

737

738

739 Supplementary Figure 3. Validation of prognostic genes of stromal/immune DEGs

740 for STAD in GEO database.

741  Relationship between stromal/immune scores obtained by ESTIMATE  algorithm and 742   overall survival   (A-B). Prognostic genes of stromal/immune DEGs for STAD  from  743 TCGA and GEO cohorts and the shared genes (C-D).

744

745

746	Supplementary Figure 4. The bar plot (A) and heat map (B) of 22 infiltrating

747	immune cells in STAD and controls estimated by CIBERSORT algorithm.
